# A comprehensive dataset of photonic features on spectral converters for energy harvesting

**DOI:** 10.1038/s41597-023-02827-3

**Published:** 2024-01-08

**Authors:** Rute A. S. Ferreira, Sandra F. H. Correia, Petia Georgieva, Lianshe Fu, Mário Antunes, Paulo S. André

**Affiliations:** 1 Department of Physics and CICECO—Aveiro Institute of Materials, University of Aveiro, 3810-193 Aveiro, Portugal; 2 Instituto de Telecomunicações, University of Aveiro, 3810-193 Aveiro, Portugal; 3 Departament of Electronics, Telecommunications and Informatics, Institute of Electronics and Informatics Engineering of Aveiro (IEETA), University of Aveiro, 3810-193 Aveiro, Portugal; 4 Departament of Electronics, Telecommunications and Informatics, University of Aveiro, 3810-193 Aveiro, Portugal; 5 Department of Electrical and Computer Engineering and Instituto de Telecomunicações, Instituto Superior Técnico, Universidade de Lisboa, 1049-001 Lisbon, Portugal

**Keywords:** Devices for energy harvesting, Electronic devices

## Abstract

Building integrated photovoltaics is a promising strategy for solar technology, in which luminescent solar concentrators (LSCs) stand out. Challenges include the development of materials for sunlight harvesting and conversion, which is an iterative optimization process with several steps: synthesis, processing, and structural and optical characterizations before considering the energy generation figures of merit that requires a prototype fabrication. Thus, simulation models provide a valuable, cost-effective, and time-efficient alternative to experimental implementations, enabling researchers to gain valuable insights for informed decisions. We conducted a literature review on LSCs over the past 47 years from the Web of Science^TM^ Core Collection, including published research conducted by our research group, to gather the optical features and identify the material classes that contribute to the performance. The dataset can be further expanded systematically offering a valuable resource for decision-making tools for device design without extensive experimental measurements.

## Background & Summary

**Fig. 1:**
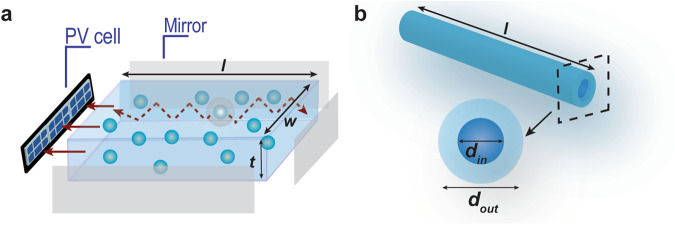
Luminescent solar concentrator concept. Scheme of (**a**) planar and (**b**) fiber-based LSC with dimensions: (*l* – length, *w* – width, *t* – thickness, *d*_*in*_ – inner diameter, *d*_*out*_ – outer diameter) in the hollow-core configuration. In this case, the doped layer is in the core (*d*_*in*_). The 4 edges of the planar LSCs may be coupled to PV cells or mirrors (or reflective tapes).

The luminescent solar concentrator (LSC) concept (Fig. 1a) dates from the late 70 s^1,2^, but major advances occurred over the last twenty years. Nowadays, LSCs are seen as an urban architecture strategy to integrate solar-harvesting devices into buildings, Fig. 1a,b^3^. This was greatly fostered by the introduction of the Zero-Energy Building (ZEB) concept and related United Nations and European Union directives^4–6^. The implementation of ZEBs implies an optimized use of renewable energy sources which draws attention to solutions that may easily contribute to the energy efficiency of buildings, through existing infrastructures and, thus, LSCs gained renewed importance over the last decade, with real-life demonstrators being developed and implemented (e.g. highway sound barriers^7–10^ and agrivoltaic applications^11,12^) and companies being founded (e.g. *Glass to Power*^13^, *UbiQD*^14^ and *ClearVue*^*PV*^)^15^. A step further on LSC development was the recently reported approach including additional sensing abilities to LSCs to behave as sunlight-powered optical temperature sensors^16,17^, which would make possible the optimization of heating/cooling systems without the need for additional energy-consuming sensors or systems, enabling substantial long-term benefits for society concerning energy consumption habits. Moreover, material science has evolved hugely over the last years in terms of achieving optically active materials with high absorption and conversion ability and small overlap between the absorption and emission spectra to prevent re-absorption losses (e.g. large Stokes-shift^18,19^ defined for organic molecules), which enabled the fabrication of large-area devices^18,20–29^. An LSC consists of planar waveguides that are either doped or coated with emissive materials. These materials absorb sunlight and re-emit it at distinct wavelengths that match the operating spectral region of the photovoltaic (PV) cells. The emitted light is then guided through total internal reflection towards photovoltaic cells coupled to the edges of the waveguides, where it is converted into electricity.

The optical conversion efficiency (*η*_*opt*_) is widely recognized as the primary figure of merit to evaluate the performance of LSCs. It quantifies the ratio of the generated output optical power (*P*_*out*_) to the incident optical power (*P*_*in*_), providing a measure of how effectively the materials convert incoming light into usable optical signal^19^. This figure of merit serves as a crucial benchmark for evaluating the efficacy of various approaches and optimizing LSC configurations to enhance overall efficiency.

Another parameter commonly used to quantify performance in terms of light harvesting and energy conversion is the power conversion efficiency (PCE). The PCE measures the ratio of the generated electrical power (${P}_{out}^{el}$) to P_in_, taking into account the specific characteristics of the coupled photovoltaic cell. The PCE provides a more comprehensive assessment of the LSC’s performance by considering the electrical power and the photovoltaic cell’s efficiency.

Despite enormous efforts, the improvement in these figures of merit is somewhat limited. Unless the intrinsic limitations, such as low absorption efficiency coefficient translated into weak radiation-harvesting capability, large self-absorption quantified by the spectral overlap between the emission and the absorption spectrum, and poor conversion efficiency quantified by a low emission quantum yield *η*_*yield*_ can be solved, it seems unrealistic to use these materials for solar energy conversion and to be active in climate change-related actions and substantial long-term benefits for society. In addition, self-absorption quantified by the overlap integral *OI*^30^ between the absorption and emission spectra (in some cases presented as the modified overlap integral OI*^31,32^ if normalized to the emission spectra) has been pointed out as one of the most critical aspects for the device performance^3,19,30–34^, although its quantification is available in very few works^30–32^. Nevertheless, bearing in mind the final goal of large-scale implementation in real applications, transparency and visible light transmittance are also key factors when thinking of replacing windows with such devices. Thus, a balance between the visual comfort of the building occupants and electrical output should be achieved.

In 1988, John Maddox wrote, “One of the continuing scandals of physical science is that it remains, in general, impossible to predict the structure of even the simplest crystalline solids from a knowledge of their chemical composition”^35^. While facing some evolution nowadays, predicting the crystal structure based solely on the composition remains challenging and entails high computational costs. An even more glaring example is the *a priori* prediction of materials compositions from the massive amount of produced and published experimental data, for a given set of target applications, as the rationalization of materials is exceptionally difficult.

Although several reviews on LSCs have been published over the years^3,19,33,36–47^, mostly concerning the type of luminescent materials in use and LSC configurations and applications, this dataset intends to go further and be a starting point to achieve a massive compilation of relevant features concerning optically active materials used to fabricate LSCs, which can be helpful for researchers working in the field. Also, this dataset has the potential to promote much-needed standardization in the reporting of figures of merit and characterization procedures for LSC devices, which is a concern^48,49^. By establishing consistent reporting practices, researchers and industry professionals can ensure comparability, reproducibility, and effective collaboration in the field.

## Methods

**Fig. 2:**
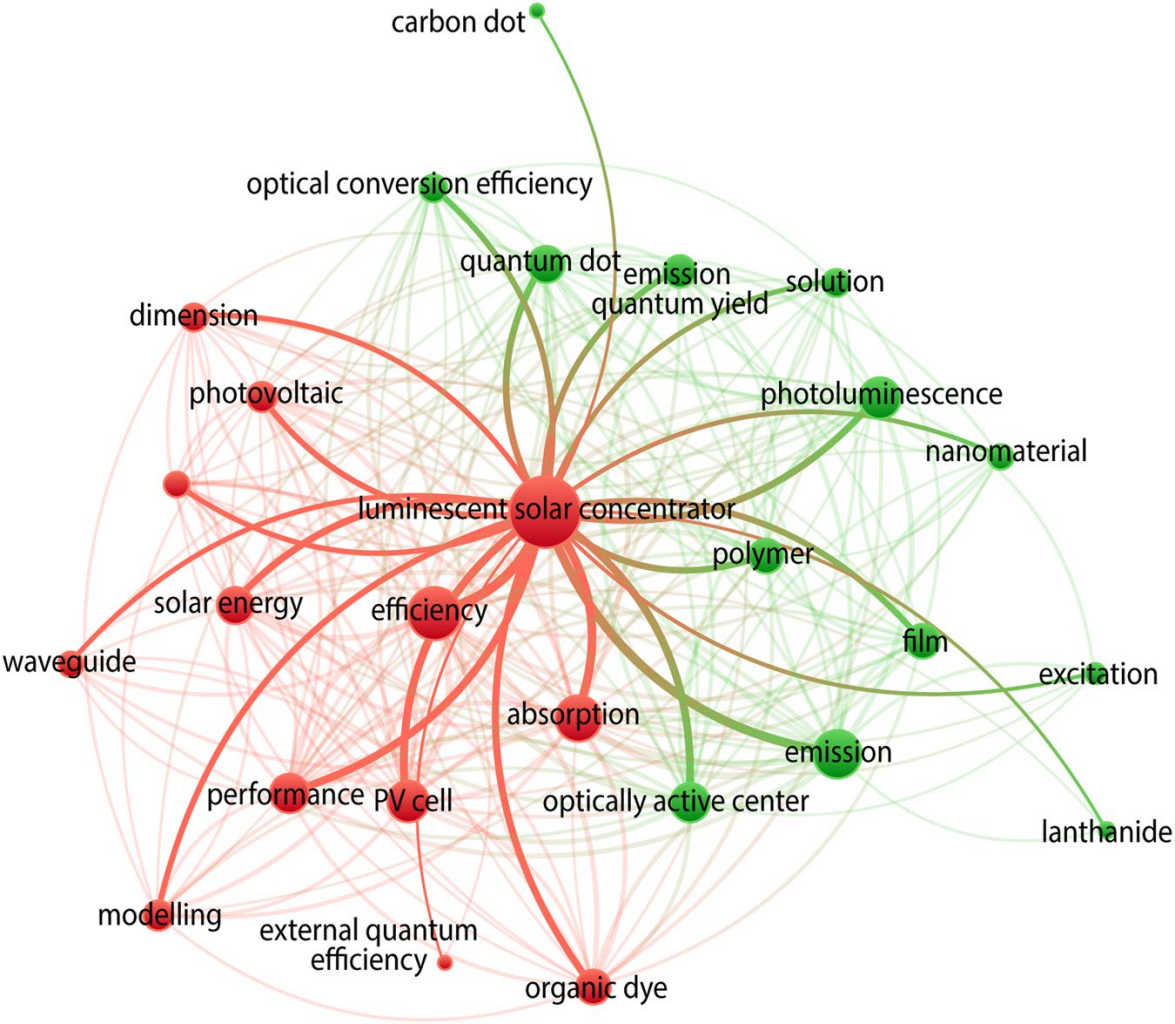
Network visualization of term occurrences extracted from abstracts and titles in 1322 publications from Web of Science^TM^ principal collection in the period 1976–2023, using ‘luminescent solar concentrator’ as the search keyword. A threshold cutoff of 10 as a number of term co-occurrence was used. The diameter of the circles is directly proportional to the number of occurrences of an indexing term, and the distance is directly proportional to the relation between them on the map (the closer two indexing terms are the more related they are). The highlighted lines represent the direct connections with the ‘luminescent solar concentrator’ term.

The dataset was collated from the community of researchers or research groups working in the development of LSC and all data sources are cited^1,11,16–18,20–30,32,50–231^. The first paper reporting the concept of LSC dates from 1976^1,2^, setting the starting point for the literature review behind the dataset. This literature review starting over the past 47 years on the field of spectral energy conversion was made using CitNetExplorer and VOSViewer tools. The sample data consisting of the information from 1474 published articles, letters, reviews, and books from the Web of Science^TM^ Core Collection containing the following citation indexes: SCI-EXPANDED, SSCI, A&HCI, CPCI-S, CPCI-SSH, ESCI, CCR-EXPANDED, and IC, using the search terms in all fields: luminescent solar concentrator or fluorescent collector or greenhouse collector in the period from 1976 to 2023, accessed on November 16, 2023. This approach has been successfully used in the field of optical sensing^232,233^. From each article, information was extracted such as authors’ names, affiliation and funding entity, the document title, keywords, abstract, and reference list, the publication citations and date, and the journal information, allowing for analyzing these fields in a multitude of parameters. We explored these fields by creating a map based on text categorical data (Fig. 2), which means that the abstracts, keywords, and titles were scanned for terms or verified whether a term is present or not (binary counting) and if it has a link with some other terms (both appearing in the same document). If the term appeared in a document, it is counted as one occurrence and if two terms appear together in the same document (co-occurrence), a link is created between them. The number of occurrences of a term was represented by the relative size of its circle. The categories, features and terms included in the dataset were chosen considering the more recurrent terms which is directly related to their relevance in the field. Also, the proximity of terms in the map was representative of how closely related they were, despite having a co-occurrence or not. Nevertheless, in some cases, a term linked with many other terms that were not related, can appear at a longer distance, being placed in the middle of all its connections. There was also the aggregation of terms that were homonyms but had different designations amongst the published papers. Based on these connections and the terms found in the research, it is possible to define two main clusters containing terms that are representative of different fields of study: one related to LSC devices and the other one related to photoluminescence spectroscopy (Fig. 2). The most relevant terms which are directly connected with the ‘luminescent solar concentrator’ term are highlighted in Fig. 2 and those are the ones addressed in the dataset here reported. The optical features and the materials classes are the links between the clusters as the indexing terms such as, for instance, emission, absorption, quantum yield, lanthanide, carbon dot, dimension, or film are shared^234,235^.

**Fig. 3:**
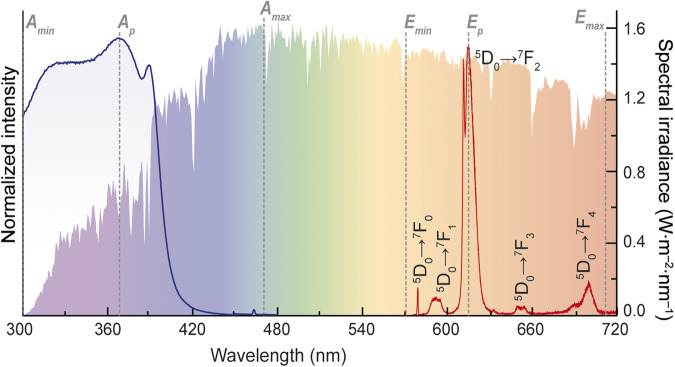
Optical features description. Excitation spectrum monitored at 612 nm and emission spectrum excited at 370 nm for a selected Eu^3+^-based organic-inorganic hybrid^110^. The shadowed area represents the Air Mass 1.5 Global solar spectrum (AM1.5 G, the spectrum generally used in terrestrial solar cell research, right *y* axis).

The dataset is composed of a description of the materials used to fabricate the LSC device, in what concerns the optically active centres type and concentration, the host material, and the processing methods. Only downshifting examples were considered as they are the vast majority of reported cases, although LSCs based on other energy conversion mechanisms such as upconversion^236,237^ or downconversion^238^ are already available. Numerical data (when available) were also manually extracted from each of the published papers to compose the table dataset. The numerical values considered for the optical characterization parameters include: i) wavelength of peak absorption or excitation (*A*_*p*_), ii) minimum wavelength of the absorption or excitation spectral band (*A*_*min*_), iii) maximum wavelength of the absorption or excitation spectral band (*A*_*max*_), iv) wavelength of peak emission (*E*_*p*_), v) minimum wavelength of the emission spectral band (*E*_*min*_), vi) maximum wavelength of the emission spectral range (*E*_*max*_), and vii) emission quantum yield (*η*_*yield*_)^239^. The general optical features are based on photoluminescence data and absorption spectra. Figure 3 illustrates the excitation and emission spectra of one reported LSC based on lanthanide ions^110^ in which the relevant parameters were assigned as follows:

(i) *A*_*p*_: wavelength at which the intensity reaches a peak value in the excitation (or absorption) spectrum measured in nanometres (nm).
(ii) *E*_*p*_: wavelength at which the emission spectrum has a maximum intensity value, measured in nm.
(iii) *A*_*min*_: low-wavelength value of the excitation (or absorption) spectrum, measured in nm. In most cases, the value of 300 nm is considered as a threshold because below this the solar irradiance is very low (∼10^−4^% of the total solar irradiance on Earth).
(iv) *A*_*max*_: high-wavelength value of the excitation (or absorption) spectrum, where the intensity exhibits significant deviation from the noise level (>5%), measured in nm.
(v) *E*_*min*_: low-wavelength value of the emission spectrum, where the intensity exhibits significant deviation from the noise level (>5%), measured in nm.
(vi) *E*_*max*_: high-wavelength value of the emission spectrum, where the intensity exhibits significant deviation from the noise level (>5%), measured in nm.

Hence, the compiled dataset is highly representative of the field, capturing a comprehensive range of optical properties and characteristics about LSCs and related materials.

The dataset is also composed of the so-called performance features like *η*_*opt*_ and PCE, which are intrinsically dependent on the dimensions of the LSC device (Fig. 1a,b), and thus this information is also provided in the dataset. By definition, *η*_*opt*_ is a measure of the ratio between the output optical power and the incident one:

1 $${\eta }_{opt}=\frac{{{P}}_{{out}}}{{{P}}_{{in}}}$$

Experimental optical measures of *P*_*out*_ and *P*_*in*_ are performed using integrating spheres or power meters to calculate *η*_*opt*_ using Eq. 1 (from this point onwards, it will be referred to as the definition equation). These parameters can also be estimated when the LSCs are coupled to a photovoltaic cell. In this scenario, the literature provides various models (expressions) that can be employed to establish a correlation between the measured electrical parameter in the photovoltaic cell and the optical power. These models offer different levels of accuracy, allowing for a more comprehensive analysis of the relationship between the two variables. Among these, Eqs. 2, 3 are frequently employed to quantify *η*_*opt*_. While Eq. 2 (higher accuracy equation) provides high accuracy by precisely incorporating the efficiency of the PV cell to correct the spectral response, Eq. 3 (lower accuracy equation) is a rougher approximation. Equation 2 is defined as follows^50^:

2 $${\eta }_{opt}=\frac{{I}_{SC}^{L}{V}_{{0}}^{L}}{{I}_{SC}{V}_{{0}}}\frac{{A}_{e}}{{A}_{s}}\frac{{\eta }_{solar}}{{\eta }_{PV}}$$

where ${{\rm{I}}}_{{\rm{SC}}}^{{\rm{L}}}$ and ${{\rm{V}}}_{0}^{{\rm{L}}}$ represent the short-circuit current and the open voltage of the photovoltaic cell coupled to the LSC, respectively (I_sc_ and V_0_ are the corresponding values of the photovoltaic cell exposed directly to solar radiation), *η*_*solar*_ is the efficiency of the photovoltaic cell relative to the total solar spectrum, *η*_*PV*_ is the efficiency of the photovoltaic cell at the LSC emission wavelengths, *A*_*e*_ is the LSC edge area, and *A*_*s*_ is the top surface area of the LSC^50^. An alternative definition, Eq. 3, is given by^170^:

3 $${\eta }_{opt}=\frac{{I}_{SC}^{L}}{{I}_{SC}}\frac{{A}_{e}}{{A}_{s}}$$

There is also more theoretical approach (theoretical equation), which considers that *η*_*opt*_ can be described by weighting all the main optical losses found in the LSC (most of them can be assessed experimentally), given by the product of the several terms in Eq. 4^240^:

4 $${\eta }_{opt}=\left(1-R\right){\eta }_{abs}{\eta }_{SA}{\eta }_{yield}{\eta }_{Stokes}{\eta }_{trap}{\eta }_{mat}$$

in which *R* is the Fresnel reflection coefficient for perpendicular incidence, *η*_*abs*_ is the ratio of photons absorbed by the emitting layer to the number of photons falling on it, *η*_*SA*_ is the self-absorption efficiency^241^, *η*_*Stokes*_ is the Stokes efficiency, *η*_*trap*_ is the trapping efficiency and *η*_*mat*_ takes into account the transport losses due to matrix absorption and scattering. This suggests that different equations yield comparable results in terms of optical conversion efficiency. However, it is worth noting that Eq. 3 is more commonly utilized.

The PCE figure of merit is obtained from experimental data using the following Eq. 5:

5 $$PCE=\frac{{P}_{out}^{el}}{{P}_{in}}=\frac{{I}_{SC}^{L}{V}_{oc}^{L}FF}{{A}_{S}{\int }_{{\lambda }_{1}}^{{\lambda }_{2}}{I}_{AM1.5G}\left(\lambda \right)d\lambda }$$

where FF is the fill factor of the photovoltaic cell. The PCE figure of merit correlates the output electrical power (which is directly dependent of the PV cell in use) to the incident optical one.

We note that the number of entries in the dataset is somewhat limited because, although the number of publications is increasing over the last 15 years (total publications ∼1500), there is a significant amount (∼80%) of published works on luminescent solar concentrators which lack performance quantification related either to *η*_*opt*_ or to PCE. This results in ∼300 published works with LSC performance quantification, matching the number of entries in the dataset.

## Data Records

The complete dataset is available at *figshare*^242^. The data is contained in an Excel file (.xlsx file, composed of 27 columns and 305 entries, which provides the data and the details of the dataset. The here presented dataset has the key to columns and units presented in the following tables, divided in two types: i) materials and the manufacturing processing categories (Table 1) and ii) numerical values considered for the optical features and electrical characterization parameters (Table 2). Table 3 describes the columns which were included in the dataset to facilitate identification and tracking of the reported LSC, such as designation, publication year and DOI of the source published work.

**Table 1:** Parameters included in the dataset related to the materials and the manufacturing processing.

Header	Explanation	Unit	Classes
type_OC	the optically active centre type	—	• dye - organic dye • Ln - lanthanide ions • QD - quantum dot • CD - carbon dot • NP - nanoparticle • polymer • a combination of the above
chemical_OC	the chemical designation of the optically active centre	—	—
concentration	the optically active centre concentration	• M • wt%	—
type_h	the material host	—	• hybrid • polymer • solvent • resin • glass
chemical_h	the chemical designation of the host material	—	—
processing	the host material processing method	—	• film • bulk • solution • fiber
method	the LSC fabrication method	—	• drop cast • spin-coating • dip-coating • spray-coating • doctor blade • liquid in container • 3D printing • scrap-coating
conditions_η_opt_	experimental measurement conditions used in the determination of the optical conversion efficiency *η*_*opt*_	—	—
conditions_PCE	experimental measurement conditions used in the determination of the power conversion efficiency *PCE*	—	—
PV cell	photovoltaic cell used in the LSC performance quantification	—	• a-Si - amorphous Silicon • c-Si - crystalline Silicon • GaAs • Perovskite • CIGS - Copper Indium Gallium Selenide • Organic • CuInSe_2_ • DSSC – dye-sensitized solar cell

**Table 2:** Parameters included in the dataset related to numerical values considered for the optical and electrical performance quantification.

Header	Explanation	Unit
dimension	LSC dimension: l × w × t for bulk/film or l × d_out_ × d_in_ (when applicable) for fibers. See Fig. 1a,b for clarification.	cm
*A*_*p*_	wavelength value for the peak absorption	nm
*A*_*min*_	minimum wavelength value for the absorption spectral band	nm
*A*_*max*_	maximum wavelength value for the absorption spectral band	nm
*E*_*p*_	wavelength value for the peak emission	nm
*E*_*min*_	minimum wavelength value for the emission spectral band	nm
*E*_*max*_	maximum wavelength value for the emission spectral band	nm
*OI*	overlap integral between absorption and emission bands	^a^
OI*	overlap integral between absorption and emission bands normalized to the emission one	^a^
*η*_*yield*_	emission quantum yield	%
*η*_*opt*_	optical conversion efficiency	%
*PCE*	power conversion efficiency	%

^a^Absolute values.

**Table 3:** Other information provided in the dataset related to the listed devices.

Header	Explanation
designation	designation used to describe the LSC: optical centre – host
year	the publication year of the source paper from which the data is obtained
DOI	the source paper DOI, allowing for easy identification and citation

## Technical Validation

To ensure data integrity and quality, only data extracted from published works in SCI-indexed journals were considered. The data related with spectroscopic features (emission and absorption/excitation) were either taken directly from the text when the figures were fully described or extracted from presented graphs (spectra), which may cause some value misreading, inducing an estimated deviation of ±10 nm. For numerical data (*OI*, OI*, *η*_*yield*_, *η*_*opt*_ and PCE), the values were extracted from the main text as reported by the authors. In what concerns the experimental data, the spectroscopic data presents the deviation associated with the measuring equipment, which is typically around 2 nm. For the *η*_*yield*_, it is important to note that the values are typically within a 10% error range, as typically stated by the manufacturer of the integrating spheres apparatus, probably related with detector sensitivity limitations and software calculations.

The error associated with the calculated values of *η*_*opt*_ and PCE was estimated using the error propagation method, which generally induces a relative error of *Δη*_*opt*_/*η*_*opt*_ and *ΔPCE*/*PCE* below 5%. The *η*_*opt*_ associated error is given by:

6 $$\begin{array}{lll}{(\Delta {\eta }_{opt})}^{2} & = & {\left(\frac{d{\eta }_{opt}}{d{I}_{sc}^{L}}\Delta {I}_{sc}^{L}\right)}^{2}+{\left(\frac{d{\eta }_{opt}}{d{V}_{0}^{L}}\Delta {V}_{0}^{L}\right)}^{2}+{\left(\frac{d{\eta }_{opt}}{d{A}_{e}}\Delta {A}_{e}\right)}^{2}+{\left(\frac{d{\eta }_{opt}}{d{\eta }_{solar}}\Delta {h}_{solar}\right)}^{2}\\  &  & +{\left(\frac{d{\eta }_{opt}}{d{I}_{sc}}\Delta {I}_{sc}\right)}^{2}{\left(\frac{d{\eta }_{opt}}{d{V}_{0}}\Delta {V}_{0}\right)}^{2}+{\left(\frac{d{\eta }_{opt}}{d{A}_{s}}\Delta {A}_{s}\right)}^{2}+{\left(\frac{d{\eta }_{opt}}{d{\eta }_{PV}}\Delta {\eta }_{PV}\right)}^{2}\\  & = & {\left(\frac{{V}_{0}^{L}{A}_{e}{\eta }_{solar}}{{I}_{sc}{V}_{0}{A}_{s}{\eta }_{PV}}\Delta {I}_{sc}^{L}\right)}^{2}+{\left(\frac{{I}_{sc}^{L}{A}_{e}{\eta }_{solar}}{{I}_{sc}{V}_{0}{A}_{s}{\eta }_{PV}}\Delta {V}_{0}^{L}\right)}^{2}+{\left(\frac{{I}_{sc}^{L}{V}_{0}^{L}{\eta }_{solar}}{{I}_{sc}{V}_{0}{A}_{s}{\eta }_{PV}}\Delta {A}_{e}\right)}^{2}\\  &  & +{\left(\frac{{I}_{sc}^{L}{V}_{0}^{L}{A}_{e}}{{I}_{sc}{V}_{0}{A}_{s}{\eta }_{PV}}\Delta {\eta }_{solar}\right)}^{2}+{\left(-\frac{{I}_{sc}^{L}{V}_{0}^{L}{A}_{e}{\eta }_{solar}}{{\left({I}_{sc}\right)}^{2}{V}_{0}{A}_{s}{\eta }_{PV}}\Delta {I}_{sc}\right)}^{2}\\  &  & +{\left(-\frac{{I}_{sc}^{L}{V}_{0}^{L}{A}_{e}{\eta }_{solar}}{{I}_{sc}{\left({V}_{0}\right)}^{2}{A}_{s}{\eta }_{PV}}\Delta {V}_{0}\right)}^{2}+{\left(-\frac{{I}_{sc}^{L}{V}_{0}^{L}{A}_{e}{\eta }_{solar}}{{I}_{sc}{V}_{0}{\left({A}_{s}\right)}^{2}{\eta }_{PV}}\Delta {A}_{s}\right)}^{2}\\  &  & +{\left(-\frac{{I}_{sc}^{L}{V}_{0}^{L}{A}_{e}{\eta }_{solar}}{{I}_{sc}{V}_{0}{A}_{s}{\left({\eta }_{PV}\right)}^{2}}\Delta {\eta }_{PV}\right)}^{2}\end{array}$$

where $\Delta {I}_{sc}^{L}=\Delta {I}_{sc}=1{0}^{-10}A$ and $\Delta {V}_{0}^{L}=\Delta {V}_{0}=1{0}^{-3}V$ (assuming an usual multimeter, as the 2400 SourceMeter SMU Instruments, Keithley), Δ*η*_*solar*_ = Δ_*PV*_ = 0.01 and Δ*A*_*e*_ and Δ*A*_*s*_ account for the error in measuring the LSC dimensions, which can be done using a measuring tape/ruler or a caliper with a 5 × 10^−4^ or 5 × 10^−5^ m error, respectively.

The *PCE* associated error is given by:

7 $$\begin{array}{lll}{\left(\Delta PCE\right)}^{2} & = & {\left(\frac{dPCE}{d{I}_{sc}^{L}}\Delta {I}_{sc}^{L}\right)}^{2}+{\left(\frac{dPCE}{d{V}_{0}^{L}}\Delta {V}_{0}^{L}\right)}^{2}\\  &  & +{\left(\frac{dPCE}{d{\int }_{{\lambda }_{1}}^{{\lambda }_{2}}{I}_{AM1.5G}\left(\lambda \right)d\lambda }\Delta {\int }_{{\lambda }_{1}}^{{\lambda }_{2}}{I}_{AM1.5G}\left(\lambda \right)d\lambda \right)}^{2}+{\left(\frac{dPCE}{d{A}_{S}}\Delta {A}_{s}\right)}^{2}\\  & = & {\left(\frac{{V}_{0}^{L}FF}{{A}_{S}{\int }_{{\lambda }_{1}}^{{\lambda }_{2}}{I}_{AM1.5G}\left(\lambda \right)d\lambda }\Delta {I}_{sc}^{L}\right)}^{2}+{\left(\frac{{I}_{sc}^{L}FF}{{A}_{S}{\int }_{{\lambda }_{1}}^{{\lambda }_{2}}{I}_{AM1.5G}\left(\lambda \right)d\lambda }\Delta {V}_{0}^{L}\right)}^{2}\\  &  & +{\left(\frac{{I}_{sc}^{L}{V}_{0}^{L}FF}{{A}_{S}{\left({\int }_{{\lambda }_{1}}^{{\lambda }_{2}}{I}_{AM1.5G}\left(\lambda \right)d\lambda \right)}^{2}}\Delta {\int }_{{\lambda }_{1}}^{{\lambda }_{2}}{I}_{AM1.5G}\left(\lambda \right)d\lambda \right)}^{2}\\  &  & +{\left(\frac{{I}_{sc}^{L}{V}_{0}^{L}FF}{{\left({A}_{S}\right)}^{2}{\int }_{{\lambda }_{1}}^{{\lambda }_{2}}{I}_{AM1.5G}\left(\lambda \right)d\lambda }\Delta {A}_{S}\right)}^{2}\end{array}$$

where $$\Delta {\int }_{{\lambda }_{1}}^{{\lambda }_{2}}{I}_{AM1.5G}(\lambda )d\lambda =0.01\;W\cdot {m}^{-2}$$.

## Usage Notes

The dataset presented in this work intends to be a pivotal resource for researchers and engineers working on the field of optical materials for down-shifting conversion for building-integrated photovoltaics. This comprehensive dataset is suitable for data driven analysis and models that may predict the efficiency of new LSCs without extensive experimental measurements. It can be continuously expanded and augmented in the future, offering the opportunity for data mining and may serve as training data for ML models.

## Data Availability

We hereby declare that no custom code was employed in the production or analysis of the data presented in this work.
